# The Comparative Toxicogenomics Database facilitates identification and understanding of chemical-gene-disease associations: arsenic as a case study

**DOI:** 10.1186/1755-8794-1-48

**Published:** 2008-10-09

**Authors:** Allan P Davis, Cynthia G Murphy, Michael C Rosenstein, Thomas C Wiegers, Carolyn J Mattingly

**Affiliations:** 1 Department of Bioinformatics, The Mount Desert Island Biological Laboratory, Salisbury Cove, Maine 04672 USA

## Abstract

**Background:**

The etiology of many chronic diseases involves interactions between environmental factors and genes that modulate physiological processes. Understanding interactions between environmental chemicals and genes/proteins may provide insights into the mechanisms of chemical actions, disease susceptibility, toxicity, and therapeutic drug interactions. The Comparative Toxicogenomics Database (CTD; ) provides these insights by curating and integrating data describing relationships between chemicals, genes/proteins, and human diseases. To illustrate the scope and application of CTD, we present an analysis of curated data for the chemical arsenic. Arsenic represents a major global environmental health threat and is associated with many diseases. The mechanisms by which arsenic modulates these diseases are not well understood.

**Methods:**

Curated interactions between arsenic compounds and genes were downloaded using export and batch query tools at CTD. The list of genes was analyzed for molecular interactions, Gene Ontology (GO) terms, KEGG pathway annotations, and inferred disease relationships.

**Results:**

CTD contains curated data from the published literature describing 2,738 molecular interactions between 21 different arsenic compounds and 1,456 genes and proteins. Analysis of these genes and proteins provide insight into the biological functions and molecular networks that are affected by exposure to arsenic, including stress response, apoptosis, cell cycle, and specific protein signaling pathways. Integrating arsenic-gene data with gene-disease data yields a list of diseases that may be associated with arsenic exposure and genes that may explain this association.

**Conclusion:**

CTD data integration and curation strategies yield insight into the actions of environmental chemicals and provide a basis for developing hypotheses about the molecular mechanisms underlying the etiology of environmental diseases. While many reports describe the molecular response to arsenic, CTD integrates these data with additional curated data sets that facilitate construction of chemical-gene-disease networks and provide the groundwork for investigating the molecular basis of arsenic-associated diseases or toxicity. The analysis reported here is extensible to any environmental chemical or therapeutic drug.

## Background

Environmental chemicals are postulated to play a critical role in the etiology of many human diseases [1-4]. To understand the impact of environmental chemicals on human health, we have developed the Comparative Toxicogenomics Database (CTD) [5,6]. CTD curates and integrates toxicogenomic data from vertebrates and invertebrates, including 124,000 chemicals, 2.6 million gene and protein sequences and their associated Gene Ontology (GO) [7] and KEGG pathway annotations [8], 128,000 taxa and 6,300 human diseases to produce a unique resource for the cross-species analysis of chemicals, genes and proteins, diseases, and their complex interactions. Biocurators at CTD curate three types of data from the scientific literature: (1) chemical-gene/protein interactions, (2) chemical-disease relationships, and (3) gene/protein-disease relationships. These data are curated in a structured format using controlled vocabularies and ontologies for chemicals, genes/proteins, diseases, molecular interactions, and organisms. Curated gene-disease relationships in CTD are integrated with data from the Online Mendelian Inheritance in Man (OMIM) database [9]. Over 110,000 molecular interactions involving 3,700 chemicals and 13,200 genes from 260 species have been curated to date. CTD also contains curated data for more than 5,700 and 2,000 gene-disease and chemical-disease relationships, respectively. CTD provides a unique perspective about chemical-gene-disease relationships by integrating data curated from the scientific literature. All interactions are linked to the original publications, allowing users to access the source data for specific details about corresponding experiments (e.g., dose-response, assay, tissue).

To demonstrate the scope and utility of the database for developing hypotheses about chemical-gene/protein-disease networks in humans, we analyzed the CTD arsenic data set as a test case. Arsenic is a global environmental toxicant, and its harmful effects can occur at very low exposure levels. It is a naturally occurring element, but is also a contaminant from industrial pollution, coal combustion, mining, metal processing, and the use of pesticides and fertilizers [10]. The potential health costs of chronic arsenic exposure are staggering, with over 500 million people at risk of exposure to contaminated water in eastern India and Bangladesh alone [11]. In certain regions of the United States, contaminated well-water is a significant health concern due to arsenic concentrations far exceeding the guidelines established by the Environmental Protection Agency [12]. Despite the diverse and serious health implications of arsenic exposure, its molecular mechanisms of action remain largely unknown, thereby hampering efforts to more accurately predict and treat associated toxicities.

Here we analyze our curated arsenic-gene interaction data and illustrate how CTD can be used to predict both arsenic-associated diseases and the impact of arsenic exposure on molecular functions and gene networks. Henceforth, "arsenic" is used to describe all arsenic containing compounds and the term, "gene" includes all forms of the gene (e.g., mRNA, protein) unless otherwise stated.

## Methods

### Curation process

#### Chemical-gene interaction

Candidate references for curation in CTD are collected by querying PubMed [13] for the co-occurrence of relevant gene and chemical terms. Controlled vocabulary terms from the National Library of Medicine's Medical Subject Headings (MeSH) [14] are used to search titles and abstracts. For the arsenic literature corpus, we initially queried PubMed with the terms "gene" and "arsenic" or "arsenical" as strings and MeSH-tagged terms. Since MeSH is a hierarchical vocabulary, use of MeSH as a query term descriptor allows retrieval of references with "child" chemical terms for arsenicals (e.g., sodium arsenite). Arsenic and arsenicals were both used because they reside in distinct hierarchical paths in the MeSH vocabulary. Among the 572 papers identified, 330 published between 2001 and 2007 were reviewed for curation; since then, additional papers describing arsenic-gene interactions have also been added to the database. Although this corpus did not include all references describing arsenic-gene interactions, it represented the most recent references analyzing molecular mechanisms of arsenic compounds. Scientific curators read abstracts or full-text articles and recorded all interactions between arsenic and a gene or protein. Curated data included: PubMed identification number, molecular interaction (using CTD curation action codes described below), interacting chemical, interacting gene, and the species in which the interaction occurred. Curators used the most specific chemical term possible from the CTD chemical vocabulary (see CTD controlled vocabularies below). In cases where only generic terms like "arsenic" were used in the abstract, the full text was consulted to determine the specific compound used (e.g., sodium arsenite). Biocurators do not manipulate, standardize, or normalize data from publications. Interactions reflect the authors' own conclusions. In cases in which authors describe microarray experiments, biocurators adhere to the authors' stated levels of significance when curating the data. Consequently, it is possible to find contradictory data in CTD, which reflect differences in experimental details and conclusions from different manuscripts. Users can explore these discrepancies by accessing the original papers associated with each interaction. Biocurators collect data from both the main text and supplementary files for a paper when appropriate or possible.

### Curated interaction analysis

All curated interaction data in CTD are publicly available. The arsenic analysis reported here is based on data downloaded and analyzed in March 2008. Prior to analysis, the data set was first manually processed as follows:

1. Curated interactions for arsenic compounds were combined. Interactions for both arsenic and arsenicals were retrieved using the CTD Curated Interaction Query [15]. The query was a "contains" search using the term "arsenic" in the Chemical query box and "ANY" as the chemical-gene interaction type. This query retrieved all curated interactions for arsenic, arsenicals, and all child terms.

2. "No effect" interactions were removed. In addition to capturing chemical-gene interactions, curators capture "no effect" results reported in the literature, such as "arsenic trioxide does not affect the phosphorylation of protein A." To avoid artificially inflating the number of interactions involving arsenic or falsely associating arsenic with a gene, all "no effect" interactions were removed from the data set.

3. Binary interactions were identified. A curated interaction in CTD must include at least one chemical and one gene (binary interaction); however, complex interactions describing more than two molecules may also be captured. For example, the following two interactions were curated from the same paper: "arsenite results in increased expression of TP53 mRNA" and "alpha-tocopherol inhibits the reaction [arsenite results in increased expression of TP53 mRNA]." The first interaction is binary and describes the core data (arsenite exposure leads to up-regulated TP53 mRNA). The second interaction is complex in that it reiterates the core data and then qualifies it with additional information. To avoid inclusion of duplicate interactions from a single reference, only binary interactions were retained in the data set.

### CTD controlled vocabularies

Several controlled vocabularies and ontologies are used to construct chemical-gene interactions and chemical- and gene-disease relationships, allowing data to be captured consistently by different curators, retrieved more easily and reproducibly by users, and integrated with other databases. Biocurators use official terms (gene symbol, chemical name, or disease name) from standardized sources (below). CTD is updated monthly, using the most current release of all external databases that are integrated with CTD (see "Data Status" link on CTD homepage for specific release versions). If an official term undergoes a nomenclature change, CTD implements that same change. Consequently, CTD is synchronized regularly with its external data sources.

1. Chemicals. The CTD chemical vocabulary derives from a modified subset of the chemicals and supplementary concepts in the "Drugs and Chemicals" category of MeSH.

2. Chemical qualifiers. A chemical term can be qualified with "analog" or "metabolite" to describe a chemical derivative (e.g., arsenic disulfide analog or arsenic metabolite).

3. Diseases. The CTD disease vocabulary uses terms from both MeSH and OMIM. To utilize both sources, we first integrated OMIM disease names (a flat list) into the MeSH disease hierarchy. Most OMIM diseases easily mapped to a single equivalent term from MeSH (*e.g*., OMIM "lung cancer" maps directly to MeSH "lung neoplasm"). Some OMIM disease names were mapped to multiple MeSH terms when a single equivalent term was not available (*e.g*., OMIM "chronic myeloproliferative disorder with eosinophilia" was most accurately mapped to two MeSH terms: "myeloproliferative disorders" and "eosinophilia"). This mapping preserved individual diseases but rooted them in a hierarchical vocabulary, allowing a user to cluster what might at first appear to be unrelated diseases (*e.g*., "Papillon-Lefevre syndrome" and "multiple café-au-lait spots") via a more general parent disease (*e.g*., "skin diseases").

4. Genes. CTD uses official gene symbols and names from the National Center for Biotechnology Information's (NCBI) Entrez-Gene database [16].

5. Gene qualifiers. A gene symbol can be qualified with any of 15 gene qualifier terms developed at CTD (*e.g*., "DNA," "promoter," "mRNA," "protein," etc.) to specify any aspect of a gene. Henceforth, we use the word "gene" in this paper to collectively refer to any aspect of a gene, including its product.

6. Action codes. CTD curators developed a hierarchical vocabulary of 50 diverse terms (*e.g*., "binding," "phosphorylation," "activity," "transport," "methylation") to describe the specific molecular interaction between any chemical and gene. A complete list of action codes and their definitions is available via the Help link for interactions on CTD glossary page and gene, interaction, and reference query forms.

7. Organisms. The CTD organism vocabulary consists of the Eumetazoa portion (vertebrates and invertebrates) of the NCBI Taxonomy database [17].

### Gene function and network analysis

GO and KEGG pathway data are integrated with CTD gene data. Annotations were downloaded for arsenic-interacting genes using the CTD batch query tool. Network analyses were performed using the Ingenuity Pathways Analysis (Ingenuity Systems) software.

### Disease analysis

There are two sources for direct disease relationships in CTD: curation of chemical-disease and gene-disease relationships from the literature, and integration of gene-disease relationships from the OMIM database [9]. Disease relationships in CTD may be direct or inferred. Direct relationships are established by CTD curation and imported from OMIM. Inferred relationships are generated computationally by integrating curated chemical-gene data with curated gene-disease data. For example, if Chemical A interacts with Gene B (via a curated chemical-gene interaction), and independently Gene B is associated with Disease C (via a curated gene-disease molecular mechanism or biomarker relationship), then Chemical A is said to have an inferred relationship with Disease C (inferred via Gene B). Disease relationships are clearly labeled as either direct or inferred in CTD. Of the 1,456 genes that interact with arsenic, 424 genes have a direct mechanism or biomarker relationship with 516 unique diseases. To identify whether there was a pattern among these 516 diseases, the diseases were rooted to their top-leveled MeSH terms and clustered into 26 categories. For example, type 1 diabetes mellitus is associated with three paths of the MeSH disease hierarchy that ultimately are rooted to three top-level disease categories: metabolic diseases, endocrine system diseases, and immune system diseases. Consequently, any arsenic-interacting gene directly associated with type 1 diabetes mellitus is counted in all three of those disease categories. The final distribution pattern for these disease categories was compared against the disease category distribution pattern for all disease-associated genes in CTD. A Pearson's chi-square test comparing arsenic associated diseases (derived from 424 arsenic-interacting genes) indicated that these associations were highly significant (p-value < 0.0001).

## Results

A curated data set for arsenic was analyzed to illustrate the scope of CTD and to highlight potential applications for understanding mechanisms of chemical actions and potential links to human diseases. This analysis included 2,738 curated interactions between 21 arsenic compounds and 1,456 genes and proteins in 17 species. These data were extracted from 280 papers published between 2001–2007.

### Arsenic compounds

**Figure 1:**
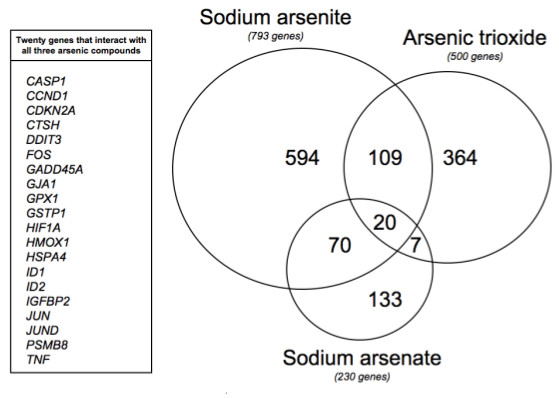
**Venn diagram of arsenic-interacting genes**. Sodium arsenite, arsenic trioxide, and sodium arsenate interact with 793, 500, and 230 genes, respectively. The 20 genes common to all three arsenic compounds are listed.

Sodium arsenite, arsenic trioxide, and sodium arsenate are the three most frequently curated arsenic compounds in CTD, interacting with 793, 500, and 230 genes, respectively (Table 1). Despite the complex chemistry and biotransformation of arsenic compounds, many (e.g., sodium arsenite and arsenic trioxide) are metabolized into a common reactive arsenite ion [18]. To determine whether different arsenic compounds affect similar genes, we compared the interacting gene sets for these top three arsenic compounds. Collectively, sodium arsenite, arsenic trioxide, and sodium arsenate had curated interactions with 1,297 genes, accounting for 89% of the total genes analyzed in this arsenic study. Among these genes, 206 overlapped with at least two compounds, with the least overlap found between sodium arsenate and arsenic trioxide (Figure 1). Twenty genes interacted with all three arsenic compounds. These data indicate that arsenic compounds share some common molecular activity, although the overall biological effects or the molecular networks they invoke may be compound-specific. Analysis of all 1,456 genes associated with all 21 arsenic compounds accounts for primary and secondary targets of arsenic and its metabolites. This approach may reasonably mimic a biological response to arsenic. It is the combination of primary, secondary and metabolite effects that contribute to the overall response to an exposure.

**Table 1:** Arsenic compounds with curated gene interactions

**Arsenic Compounds**	**Genes^1^**
Sodium arsenite	793
Arsenic trioxide	500
Sodium arsenate	230
Arsenic	127
Arsenite	115
Arsenic disulfide	54
Dimethylarsinous acid	28
Gallium arsenide	19
Cacodylic acid	17
Monomethylarsonic acid	17
Arsenates	16
Arsenic trichloride	8
Trimethylarsine oxide	8
Dimethylarsine	7
Methylarsine oxide	6
Arsenic trisulfide	5
Arsenic acid	4
Oxophenylarsine	3
Arsenicals	2
Arsenous acid	1
Lewisite	1

^1^Some genes interact with more than one arsenic compound

### Molecular actions of arsenic

Curated chemical-gene interactions in CTD indicated that arsenic has a broad range of molecular effects (Table 2). The most common arsenic interaction involved effects on transcription: 810 genes were up-regulated and 652 were down-regulated. Arsenic also affected protein activity, phosphorylation status, degradation, localization, secretion, metabolism, and stability. Promoter methylation and gene mutagenesis effects of arsenic were also described. Molecular interactions involving effects of proteins on arsenic included resistance/susceptibility to arsenic, arsenic metabolism, arsenic transport, and arsenic binding.

**Table 2:** Types of curated arsenic-gene interactions

**Arsenic-to-Gene Molecular Interactions**	**Genes^1^**
mRNA up-regulated	810
mRNA down-regulated	652
Protein up-regulated	97
mRNA/protein altered^2^	82
Protein activity	78
Protein down-regulated	49
Protein phosphorylation	39
Protein cleavage/degradation	14
Protein localization	14
Protein secretion	8
Promoter methylation	7
Protein metabolism	6
mRNA/protein stabilization	4
Gene mutagenesis	4
	
**Gene-to-Arsenic Molecular Interactions**	**Genes^1^**

Arsenic resistance/susceptibility	43
Arsenic transport	5
Arsenic metabolism	5
Protein binds arsenic	5

^1 ^Some genes are counted in more than one interaction category

^2 ^Molecule (mRNA or protein) and/or direction (up or down-regulation) were not specified

### Arsenic-interacting genes

Gene Ontology (GO) annotations and KEGG pathway data are integrated in CTD to provide additional biological context to genes and chemical effects. GO annotates gene products in terms of their associated molecular function, biological process, and cellular component [7]. KEGG is a public resource that presents information about molecular reaction networks [8]. Arsenic-interacting genes were evaluated using GO, KEGG, and Ingenuity Pathway Analysis (IPA) to gain insight into the biological functions and molecular networks that may be altered by arsenic exposure.

The top 25 genes with curated arsenic interactions included several transcription factors (JUN, MYC, TP53, EGR1, ESR1, DDIT3, FOS) and regulators of cell cycle, cell proliferation, and apoptosis (BCL2, CCND1, CDKN2A, VEGFA, CDNK1A, GADD45A, BAX), suggesting that a dramatic cellular response may be induced by arsenic exposure (Table 3). Seven of these genes (HMOX1, CCND1, CDKN2A, GADD45A, GSTP1, DDIT3, FOS) interact with multiple arsenic compounds (Figure 1).

**Table 3:** Most frequently curated arsenic-interacting genes

**No. ** **Curated ** **Interactions**	**No. ** **Interacting ** **As ** **Compounds^1^**	**Gene**	**Molecular ** **Function^2^**	**Biological ** **Process^2^**
44	9	*HMOX1*	Heme oxygenase	Heme oxidation
21	3	*BCL2*	Protein binding	Regulation of apoptosis
21	6	*JUN*	Transcription factor	Regulation of transcription, DNA-dependent
19	6	*ABCC2*	ATP-binding transporter	Transport
19	6	*MAPK1*	MAP kinase	Protein amino acid phosphorylation
18	6	*MAPK3*	MAP kinase	Protein amino acid phosphorylation
17	5	*ABCC1*	ATP binding	Transport
17	4	*CCND1*	CDK regulator	Regulation of cell cycle
17	5	*CDKN2A*	Kinase	Cell cycle arrest; regulation of cyclin dependent protein kinase activity
16	6	*AS3MT*	S-adenosylmethionine-dependent methyltransferase	Arsonoacetate metabolism; toxin metabolism
16	3	*MYC*	Transcription factor	Regulation of transcription, DNA-dependent
15	3	*VEGFA*	Growth factor	Angiogenesis; anti-apoptosis; cell cycle
14	4	*GADD45A*	Protein binding	Cell cycle arrest; DNA damage response
14	6	*NQO1*	NADPH dehydrogenase	Electron transport
14	4	*TP53*	Transcription factor	Regulation of transcription, DNA-dependent
13	5	*AFP*	Carrier	Transport
13	4	*CDKN1A*	CDK inhibitor	Cell cycle arrest
13	4	*GSTP1*	Glutathione transferase	Metabolism
13	3	*HSPA1A*	Unfolded protein binding	Protein folding; response to unfolded protein
12	5	*EGR1*	Transcription factor	Regulation of transcription, DNA-dependent
12	4	*ESR1*	Transcription factor	Regulation of transcription, DNA-dependent
12	4	*PCNA*	DNA polymerase processivity factor; DNA binding	Regulation of DNA replication
11	5	*DDIT3*	Transcription factor	Regulation of transcription, DNA-dependent
11	3	*FOS*	Transcription factor	Regulation of transcription, DNA-dependent
10	2	*BAX*	Protein binding	Regulation of apoptosis

^1 ^Out of 21 total arsenic compounds curated from the literature (see Table 1)

^2 ^Most frequently annotated GO terms are shown; complete list is available at CTD

GO and KEGG pathway annotations for all arsenic-interacting genes in CTD were evaluated and comprised 1,042 distinct molecular functions, 2,093 biological processes, 383 cellular components, and 182 pathways (Table 4). The most prominent annotated terms from each category included protein binding (GO: molecular function); transcription or signal transduction (GO: biological processes); nucleus, cytoplasm, or membrane (GO: cellular component); and MAPK signaling pathway (KEGG). Other frequently annotated processes and pathways for the 1,456 genes included apoptosis, cell cycle, cell proliferation, immune response, and cell signaling (Table 4). These processes and pathways were also implicated when using only the top 25 genes with curated arsenic interactions (Table 3), suggesting that this subset of genes may accurately represent the overall biology of genes responding to arsenic exposure.

**Table 4:** GO and KEGG annotations for arsenic-interacting genes

**GO-Molecular Function (top 10 out of 1042 total)**	**Genes^1^**
Protein binding	826
Metal ion binding	261
DNA binding	254
Nucleotide binding	243
ATP binding	203
Zinc ion binding	193
Transferase activity	181
Hydrolase activity	173
Transcription factor activity	157
Receptor activity	152
**GO-Biological Process (top 10 out of 2093 total)**	**Genes^1^**

Regulation of transcription, DNA-dependent	229
Signal transduction	227
Transcription	190
Multicellular organismal development	130
Apoptosis	121
Transport	121
Protein amino acid phosphorylation	112
Cell cycle	110
Immune response	104
Cell proliferation	88
**GO-Cellular Component (top 10 out of 383 total)**	**Genes^1^**

Nucleus	493
Cytoplasm	464
Membrane	438
Integral to membrane	295
Extracellular space	266
Extracellular region	216
Plasma membrane	176
Intracellular	156
Cytosol	121
Integral to plasma membrane	116
**KEGG Pathway (top 10 out of 182 total)**	**Genes^1^**

MAPK signaling pathway	89
Cytokine-cytokine receptor interaction	77
Focal adhesion	72
Cell cycle	56
Apoptosis	48
p53 Signaling pathway	44
Toll-like receptor signaling pathway	42
Regulation of actin cytoskeleton	41
ErbB Signaling pathway	39
Jak-STAT signaling pathway	38

^1 ^Some genes are counted in more than one annotation category

**Figure 2:**
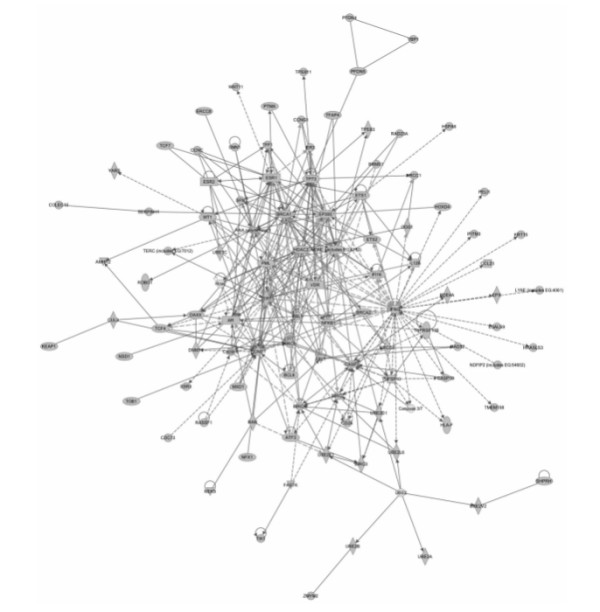
**Arsenic-responsive interactome**. Arsenic-interacting genes were evaluated for enrichment of molecular interactions. Ingenuity Pathway Analysis identified a large interaction network of 105 proteins enriched with roles in cell cycle control, apoptosis, DNA repair, and associated significantly with cancer (p < 10^-34 ^based on the hypergeometric distribution and calculated with the right-tailed Fisher's Exact Test; dotted and solid lines indicate indirect and direct interactions, respectively).

To provide a more detailed view of molecular networks potentially affected by arsenic, IPA was used to analyze arsenic-interacting genes in CTD. Analysis of the 1,376 genes with annotations in the IPA database identified a large arsenic-modulated interaction network comprising 105 proteins (Figure 2; see additional file 1). Pathway analysis showed that the network was enriched with roles in cell cycle control, apoptosis, and DNA repair, and was associated significantly with cancer. The network contained transcriptional regulators (28 proteins), enzymes (18 proteins), cytokines (4 proteins), ligand-dependent nuclear receptors (4 proteins), transmembrane receptors (3 proteins) and peptidases (2 proteins). Locations of the proteins within the network were distributed among the nucleus (52 proteins), cytoplasm (27 proteins), extracellular space (8 proteins) and plasma membrane (8 proteins). Biological implications of this network are consistent with GO, KEGG, and disease associations identified by other methods and described in this report.

### Arsenic-gene-disease associations

Curated direct chemical-gene interactions and direct gene-disease associations in CTD are integrated to create inferred chemical-disease relationships. For example, if Chemical A interacts with Gene B (via a curated chemical-gene interaction), and Gene B is associated independently with Disease C (via a curated gene-disease relationship), then CTD presents an inferred relationship between Chemical A and Disease C (inferred via Gene B). We assessed whether it was valid to use these inferred relationships to predict diseases influenced by environmental chemicals.

**Figure 3:**
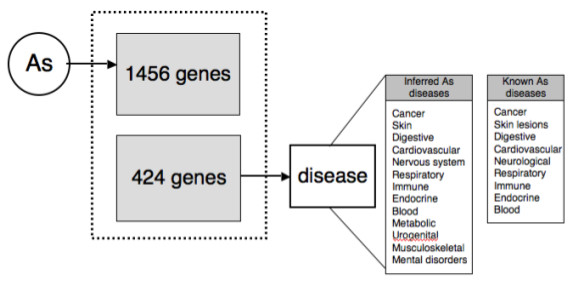
**Arsenic-gene-disease predictions**. CTD describes a molecular interaction between arsenic (As) and 1,456 genes; 424 of those genes are also directly associated with a disease. The integration of these two data sets predicts diseases that parallel those already known to be associated with arsenic exposure, underscoring the potential value and validity of these inferred relationships.

Among the genes with curated arsenic interactions in CTD, 424 have curated relationships with 516 diseases. Therefore, arsenic has inferred relationships with 516 diseases in CTD. Using the hierarchical disease vocabulary in CTD, these diseases were clustered into 26 general categories (see Methods). The most common disease categories associated with arsenic included neoplasms, nervous system diseases, skin diseases, digestive system diseases, metabolic disorders, and immune system diseases (Table 5). Notably, many of these diseases were corroborated in the literature as being associated with arsenic, including malignancies (skin, lung, liver, kidney and bladder cancer), neurological defects (peripheral neuropathy and cognitive impairment), skin lesions, diabetes, and many others (Figure 3) [19-21]. This corroboration supported the value of using CTD-inferred disease relationships to develop novel hypotheses about the health effects of exposure to environmental chemicals.

**Table 5:** Diseases associated with arsenic-interacting genes

**Disease Category**	**Genes^1^**
Neoplasms	177
Nervous system	147
Skin	140
Digestive system	97
Metabolic	86
Immune system	70
Disorders of environmental origin	65
Female urogenital	51
Musculoskeletal	51
Hematologic	48
Cardiovascular	45
Male urogenital	45
Endocrine system	44
Respiratory tract	42
Mental disorders	30
Eye	24
Stomatognathic (mouth, tooth)	22
Virus	21
Lymphatic	15
Connective tissue	14
Bacterial infections	9
Parasitic	8
Nutrition disorders	7
Otorhinolaryngologic (ear, nose, throat)	7
Pregnancy complications	5
Infection	2

^1 ^Some genes occur in more than one disease category

CTD provides candidate genes that may help to explain mechanisms underlying chemical-disease relationships. Cancer was the disease category most frequently associated with arsenic via curated interactions with 177 genes in CTD. These diseases were subdivided into specific types of malignancies, each with a unique set of associated genes: breast (49 genes), stomach (42 genes), leukemia (23 genes), nervous system (19 genes), lung (18 genes), colon (17 genes), bladder (15 genes), and others (Table 6). Although some of these disease associations have been documented (e.g., lung cancer), the molecular mechanisms underlying the etiologies remain largely unclear [22-24]. The inferred relationship between arsenic and lung cancer is established in CTD via curated interactions with 18 genes. Notably, IPA analysis of these genes identified a network involving 72% of these genes (AREG, BIRC5, EGFR, ERBB2, ESR1, GCLC, HMOX1, KRAS, MAP3K8, MAPK14, PIK3CA, RASSF1, TGFA). This network suggests that these genes may function coordinately and form a basis for investigating a mechanistic connection between arsenic and lung cancer. CTD enables analyses that will lead to the development of novel hypotheses about the molecular etiologies of environmental diseases.

**Table 6:** Cancers associated with arsenic-interacting genes

**Cancer**	**Genes**	**Gene List**
Breast	49	*AKAP12; AKT1; ATM; BCL2; BCL2A1; BIRC5; BRCA1; BRCA2; CASP8; CCL20; CHEK2; CXCL2; CXCL3; CYP19A1; CYP1A1; CYP1B1; EGFR; ERBB2; ESR1; ETS2; FGFR2; FOS; FST; HMOX1; HRAS; IL1B; IL6; IL8; JUN; KRAS; NFKBIA; NR2F1; NR2F6: PDGFA; PIK3CA; PIM1; PTEN; PTGS2; RAD51; RGS2; SLC22A18; SNAI2; SYNJ2; TANK; TFPI2; TFRC; TNF; TP53; TYMS*
Stomach	42	*ALDH1A3; AURKB; BDNF; BIRC2; BIRC5; BNIP3; CASP8; CASP10; CAV1; CDH1; CDKN1A; CDKN2A; CDKN2D; CHFR; CTSL; EEF1A2; ERBB2; F2R; FGFR2; FST; FYN; HMOX1; IGFBP3; IGFBP7; IL1B; IL1RN; IL6R; IRF1; KRAS; MT2A; MYC; NOS3; PIK3CA; PLAU; RBP1; RGS2: RORA; SERPINE1; SOD2; SPRR2A; TFAP2C; TIMP3*
Leukemia	23	*AKT1; AQP9; BAD; BCL2; BCR; BIRC2; BIRC3; CCND1; CD44; CEBPA; CFLAR; ETV6; F3; HOXD4; ITGAM; ITGB2; KIT; NSD1; PML: PTEN; PTPN11; RARA; TFRC*
Nervous system	19	*BRCA2; CTNNB1; EGF; ERBB2; HGF; IL1B; IL8; MET; MGMT; MMP2; MMP9; MYCN; NCOR1; MNE1; PMS2; PPARG; PTEN; TFRC; VEGFA*
Lung	18	*AREG; BIRC5; CASP8; EGFR; ERBB2; ESR1; GCLC; HMOX1; KRAS; MAP3K8; MAPK14; PHLDA2; PIK3CA; RASSF1; SLC22A18; TFRC; TGFA; TP53*
Colon	17	*AKT1; CASP8; CHEK2; EGFR; EP300; ERBB2; MLH1; MSH6; ODC1; PIK3CA; PMS2; PTGS2; SMAD7; TFRC; TGFBR2; TP53; TYMS*
Bladder	15	*BIRC3; CDKN2A; EGFR; ESR1; HRAS; KRAS; MT1A; MT2A; MT3; PRSS3; RASSF1; RB1; SRC; TFRC; TRP53*
Skin	15	*CDKN2A; DDB2; ERCC2; ERCC3; ERCC4; ERCC5; GYPA; HRAS; KRT10; MDM2; PMS2; TP53; XPA; XPC; XRCC3*
Prostate	11	*AR; BRCA2; CHEK2; ERBB2; ESR1; ICAM1; IL8; MMP9; MT2A; PTEN; VEGFA*
Liver	10	*AFP; CASP8; CTNNB1; ESR1; MAPK14; MET; PIK3CA; RARA; TP53; TRIM24*
Ovary	10	*AKT1; BIRC5; BRCA1; BRCA2; CYP19A1; EGFR; FST; MAPK1; MAPK3; PIK3CA*
Esophagus	8	*CASP8; MT1; MT1G; PTGS2; RNF6; SNAI2; TGFBR2; TRP53*
Lymphoma	7	*BCL2; BHMT; CASP10; MLH1; MYC; TFRC; TYMS*
Head and neck	6	*AREG; EGFR; MAPK1; MAPK3; TGFA; TNFRSF10B*
Thyroid	5	*HRAS; MINPP1; PTEN; TP53; TRIM24*
Kidney	3	*CYP1A1; MET; OGG1*
Pancreas	3	*BRCA2; KRAS; TP53*

## Discussion

CTD provides curated and integrated toxicological data to facilitate hypotheses about chemical-gene interaction networks and the impact of chemical exposures on human diseases. Knowledge derived from CTD has potential for predicting toxicity, identifying biomarkers of exposures, and unveiling putative therapeutic targets for diseases. Here we describe how CTD can be used to explore chemical-gene-disease associations using arsenic as a test case.

Arsenic represents a global environmental health threat and is associated with a wide variety of diseases such as cancers (skin, bladder, kidney, colon), skin lesions, neuropathies, memory defects, cardiovascular disorders (atherosclerosis, hypertension), diabetes, anemia, and respiratory stress [19-21]. Despite its global impact, the molecular basis of arsenic-induced disease is still not clear. To provide insight into these mechanisms, we have curated arsenic-gene interactions and arsenic-disease relationships reported in the literature using controlled vocabularies for chemicals, genes, organisms, molecular actions, and diseases. These vocabularies are key for allowing flexible querying, consistent retrieval, and complex data analysis. Integration of curated chemical-gene interactions with other important data sets such as diseases, sequences, GO annotations, KEGG pathways, and published references creates a unique method for generating insight into the molecular actions and potential health effects of diverse chemicals.

At the time of this analysis, CTD contained 2,738 curated interactions between 1,456 genes and 21 arsenic compounds. Since the chemistry and biotransformation of arsenic is complex and different compounds can result in similar arsenic metabolites [18], we analyzed data for all arsenic compounds collectively. We acknowledge that there are many complex issues associated with chemical exposures, such as dose, route, duration, metabolism, and developmental stage of exposure. Although these issues are not addressed directly in CTD, all curated data is hyperlinked to the original source for researchers who require those details. The predominant molecular effect of arsenic is alteration of gene transcription. Arsenic compounds also affect activity, phosphorylation status, metabolism, localization, and secretion of proteins. In addition, several proteins influenced susceptibility to arsenic, its metabolism, and its transport.

GO, KEGG pathway, and gene network analyses of arsenic-responsive genes in CTD provided broad insights into the diverse biological functions and pathways affected by arsenic. The most frequently curated gene interacting with arsenic was the heme oxidase HMOX1, a known stress response protein. HMOX1 was originally discovered as an enzyme involved in the degradation of heme, but has since been found to play a role in many other cellular processes, such as protection against noxious stimuli [25]. HMOX1 is regulated by a wide variety of stress-responsive transcription factors, including members of the heat-shock, nuclear factor-kappaB, nuclear factor-erythroid 2, and activator protein-1 transcription factor families [25]. Notably, 14 members of these families also have curated arsenic interactions in CTD (Figure 1, Table 3, and data not shown), supporting a hypothesis that arsenic may induce a diverse transcriptional cascade. Curated chemical-gene interactions in CTD may be used to develop insight into how complex interactive networks of genes and proteins are influenced by environmental chemicals.

Curated data in CTD complement data from recent microarray reports of global transcriptional effects of arsenic. Andrew et al. reported 206 genes with significantly altered expression in lymphocytes from 21 U.S. citizens exposed to arsenic-contaminated drinking-water [26]. Among those genes, 20 currently have curated arsenic interactions in CTD. The group also mapped their 206 genes to the same GO and KEGG pathway annotations that we found in our analysis of 1,456 genes, including immune response, signal transduction, apoptosis, cell cycle, cell proliferation, and JAK-STAT pathway. Similarly, Fry et al. identified three sets of genes that were highly predictive of arsenic exposure in specific human populations [27]. These arsenic-responsive sets consisted of 151, 34, and 11 genes. Notably, CTD had curated arsenic interactions for 15%, 38%, and 64% of the genes from each set, respectively, substantiating the potential value of CTD for identifying biomarkers of chemical exposure.

CTD is valuable in refining biomarker candidates and addressing questions about whether a gene is responsive to a specific chemical or representative of a more general stress response. Specifically, the breadth of chemical representation and curated data in CTD enables researchers to compare interacting genes for multiple chemicals or chemical categories. Interacting genes in CTD were compared between arsenic and other environmental chemicals in CTD, including heavy metals (1,489 genes), estradiol (1,230 genes), and dioxins (502 genes). Overlapping genes were removed, identifying 795 genes that were unique to arsenic action. CTD may be used alone or in combination with experimental methods such as microarray analysis to refine or provide additional confidence in putative biomarkers of environmental exposure.

CTD may be used to develop hypotheses about relationships between environmental chemicals and human diseases. Among the arsenic-interacting genes in CTD, 424 are independently associated with a disease. The associative structure in CTD allows inferred arsenic-disease relationships to be constructed. Many of these inferred diseases were corroborated in the literature (*e.g*., cancer, neuropathies, skin lesions, metabolic disorders, and cardiovascular diseases, amongst others), thereby supporting the curation and integration paradigms of CTD for developing testable hypotheses about chemical-disease relationships.

The molecular basis of most arsenic-induced disease is still not clear. CTD can offer inroads to discovering such molecular mechanisms. Inferred chemical-disease associations create a foundation for developing hypotheses about relationships between chemicals, genes, diseases, and the mechanisms of environmentally influenced diseases. For example, CTD users may identify genes that interact with arsenic and are independently associated with a neoplasm, such as the 18 arsenic-interacting genes associated with lung cancer (Table 6). These genes may be leveraged for experimental or epidemiological studies with specific emphasis on understanding arsenic-associated lung cancer such as evaluating polymorphisms in these genes from lung cancer patients in arsenic-exposed vs. control regions, testing the value of these genes or their products as biomarkers in exposed vs control populations, or investigating possible interactions between these proteins in lung cancer cell lines. Similarly, the literature supports a relatively new correlation between chronic arsenic exposure and diabetes [28,29]. Among the arsenic-interacting genes in CTD, 105 are associated with diabetes directly or are annotated with a GO term or KEGG pathway involved in insulin or glucose signaling or metabolism, providing a basis for examining the etiology of arsenic-associated diabetes.

Arsenic is associated with diverse diseases. This diversity may reflect the broad scope of genes that interact with arsenic (Tables 3 and 4). It is possible that alteration of proteins such as transcription factors or receptors that affect expression of many downstream genes and broad cellular processes may result in different phenotypic outcomes. Recent reports have postulated that arsenic may act as an endocrine disruptor, affecting the signaling pathways of steroid hormone receptors, and this dysregulation may contribute to the diverse nature of arsenic-associated diseases [30,31]. Interestingly, among arsenic-interacting genes in CTD, 73 genes (5%) are annotated with a GO term or KEGG pathway associated with hormone/steroid metabolism or signaling. In addition, breast cancer (a steroid hormone-associated malignancy) is associated with the largest number of arsenic-interacting genes in CTD (Table 6).

In addition to providing data for environmental chemicals, CTD also includes abundant data for therapeutic chemicals. For example, arsenic trioxide is commonly used to treat acute leukemia and other cancers [32-35]. Genes that interact with arsenic trioxide in CTD can be analyzed to look for common biological processes or pathways associated with arsenic trioxide therapy. CTD could be used to better define the molecular pathways and genes targeted by arsenic trioxide for the purpose of developing alternative therapeutic agents to circumvent chemical resistance or toxicity.

## Conclusion

CTD provides curated chemical-gene interactions and integrates this data with gene, disease, reference, GO annotation, KEGG pathway, organism, and sequence information to enhance understanding about the effects of chemicals on molecular networks and human diseases. Using arsenic as a test case, we report 2,738 molecular interactions between 21 different arsenic compounds and 1,456 genes/proteins. Analyses of these genes provide insight into the biological functions and molecular networks that are affected by exposure to arsenic. Integrating this arsenic-gene data set with a gene-disease data set yields a list of 516 putative diseases that interestingly parallel known arsenic-associated diseases categories. This work demonstrates and validates the utility of CTD for understanding the molecular networks and biological functions affected by chemicals. In this respect, CTD may be used as a tool to provide connections between chemicals, genes, and diseases that may not otherwise be apparent, and may provide the basis for testable hypotheses about the mechanisms underlying the etiology of environmental diseases. The analysis reported here for arsenic should be applicable to any environmental chemical or therapeutic drug.

## Abbreviations

CTD: Comparative Toxicogenomics Database; GO: Gene Ontology; MeSH: Medical Subject Headings; NCBI: National Center for Biotechnology Information; OMIM: Online Mendelian Inheritance of Man

## Availability and requirements

Project name: The Comparative Toxicogenomics Database; Project home page: ; Operating systems: Platform independent; Data model documentation:  Programming language: N/A; Other requirements: modern web browser; License requirement: none.

## Competing interests

The authors declare that they have no competing interests.

## Authors' contributions

APD designed and implemented the manual curation paradigm for CTD, curated and analyzed the arsenic data, and drafted the manuscript. CGM curated arsenic data from the literature. MCR designed and implemented the data architecture and web site for CTD. TCW processed and loaded curated and integrated data for CTD. CJM conceived of the study, participated in its design and coordination, performed gene network analysis, and helped draft the manuscript. All authors read and approved the final manuscript.

## Pre-publication history

The pre-publication history for this paper can be accessed here:


